# Study on the energy evolution mechanism and periodic micro-fracture of the BR under triaxial compression tests

**DOI:** 10.1038/s41598-025-27247-8

**Published:** 2025-12-08

**Authors:** Yuye Tan, Ziyi Zeng, Jiazhao Chen, Mochuan Guo, Weidong Song

**Affiliations:** 1 Key Laboratory of High-Efficient Mining and Safety of Metal Mines (Ministry of Education of China), University of Science and Technology Beijing, Beijing, 100083 China; 2 School of Resources and Safety Engineering, University of Science and Technology Beijing, Beijing, 100083 China; 3 Changchun Gold Research Institute Co.,Ltd, Beijing, China; 4 Sinosteel Mining Beijing Co Ltd, Beijing, 100083 China

**Keywords:** Backfill-encased-rock composite structure, Triaxial compression, Acoustic emission, Computed tomography, Failure stage, Visualization of damage evolution, Energy science and technology, Engineering, Natural hazards, Solid Earth sciences

## Abstract

In underground metal mining, the complex environment and in-situ stress control requirements impose increasingly strict standards on backfill mining methods. Owing to its advantages of safety, high efficiency, economy, and environmental friendliness, backfill mining has become an inevitable choice for green mining. This study focuses on the two-step stope post-backfill method, where the stability of the backfill-encased-rock (BR) composite structure—formed by backfill wrapping rock—plays a crucial role in overall stope stability.Taking the BR composite structure as the research object, with single rock and backfill as controls, this study conducted triaxial compression tests combined with acoustic emission (AE), computed tomography (CT) technology, and theoretical analysis. It revealed the stage characteristics of the stress-strain curve during BR’s deformation and failure, as well as the energy evolution and crack propagation laws in each compression stage. The results indicate that BR’s stress-strain curve undergoes six stages, while its AE evolution is divided into five stages. As confining pressure increases, BR’s peak strength rises and energy release shows regular changes; CT scanning clearly presents its porosity variation and crack propagation rules.This study clarifies BR’s failure law under high stress: its peak strength is 20%–30% lower than pure rock, and residual strength is 65%–80% higher than pure backfill. These findings reflect the synergistic mechanical effect between backfill and rock under triaxial compression, and provide important guidance for stope structure stability research in practical mining engineering.

## Introduction

**Fig. 1:**
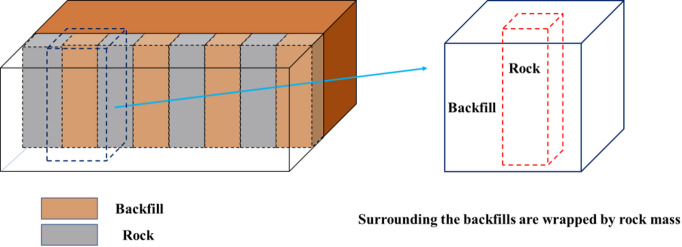
Underground structure: backfill-encased-rock.

The cut and fill stoping method has the advantages of safety, high efficiency, economy, and environmental protection. This method is the development direction and inevitable choice of green mining. Among them, two step stope and subsequent filling mining method has gradually become the preferred mining method for underground mining because of its high efficiency and degree of automation^1–4^. In this mining method, the treatment of room, pillar, and mined-out area is regarded as a whole. The typical process features are as follows: the mining pillar is first followed by cemented filling, and under the stable support frame formed by the cemented pillar, the mining room is then followed by tailings filling^5^. In the process of mining, pillar and backfill will form a structure of interleaving and wrapping each other, As shown in Fig. 1^6^. As a key part or even a permanent part in underground stope, the overall stability of this composite structure is crucial for controlling in-situ stress in stope and maintaining long-term stability of underground stope structure^7^.This study explores the failure law of the backfill-encased-rock(BR) composite structure, which provides reference for the filling method of underground structures in the future.

Mineral rock and filling physical characteristics are different, belong to different medium. In the process of underground mining, two kinds of medium are combined to form a whole and bear external load together. Therefore, it is often limited to study the mechanical properties of a certain medium or construct a single medium constitutive model, which can not accurately reflect the real situation. The existing experimental studies show that there are great differences in the bearing mechanism and damage evolution of the coupling model between single medium and multiple medium under external loads^8^.

At present, the main research methods for the mechanical properties of multiple medium coupling models are similar material physical model and numerical simulation. The physical model of similar materials is mainly to study the mechanical characteristics, stress-strain relationship, crack evolution, progressive failure mechanism, energy evolution characteristics and laws of composite structures through uniaxial and triaxial mechanical loading experiments^9–13^. Among the existing research results, the mechanical properties of the backfill-encased-rock composite structure are mainly studied as follows: through shear and triaxial compression tests, SEM observation and thermal difference analysis are conducted on the test specimens after the test, and the difference of shear parameters at the interface of BR specimens is studied^14^. The deformation characteristics and infrared radiation changes in the fracture process of the combined model of backfill and rock were studied by shear test^15–21^. Using paraffin wax as the composite structure contact zone material, the non-uniform force variation and stability of the early backfill strength contact zone were studied^22^. The stress-strain curves of specimens with different structures are summarized by comparing the results of the laboratory triaxial compression tests of a single specimen and the combined structure of surrounding rock and backfill^23^. The shear behavior of the contact surface between the cemented backfill and surrounding rock in short curing time was studied by shear test^21^.

The numerical simulation method is more economical and convenient. For the backfill-encased-rock composite structure, the research process involves a variety of media with different properties, and the methods used are mainly finite element method, discrete element, finite difference and so on^24–27^. Researchers have studied the deformation and damage characteristics and mechanical properties of composite structures by using PFC particle flow software^28^, damage constitutive model^29^, equivalent discontinuous modeling method of jointed rock mass^30^, numerical model construction^31^, and finite element based porous rock model reconstruction method^32^.

In previous studies, researchers have applied sufficient research methods and achieved satisfactory results^33–37^.However, most studies primarily focus on the failure modes of multi-material composite structures and the interaction mechanism between the two media during failure, with limited analysis on the progressive failure process of composite structures. Recent studies have further expanded the research scope of underground mining-related mechanical behaviors: in terms of overburden stability under repeated mining, relevant research has revealed the overburden failure characteristics and fracture evolution rules under repeated mining with multiple key strata control^38^; in terms of working face entry layout, studies have proposed a determination method of rational position for working face entries in the coordinated mining of section coal pillars and lower sub-layer^39^; in terms of dynamic disaster mechanism, research based on the folding catastrophe model has clarified the disaster-causing mechanism of spalling rock burst in coal mines^40^. Meanwhile, studies on backfill-rock composite structures have focused on microscale interface characteristics: Ru et al. (2025) investigated the effect of multiscale interface roughness on the shear behavior of backfill-rock specimens, revealing that interface morphological features significantly regulate shear stress transmission and failure modes^41^; they also established a shear strength prediction model for backfill-rock interfaces based on 3D morphology analysis, providing a quantitative basis for interface performance evaluation^42^. These latest studies have enriched the research system of underground mining engineering mechanics, but the progressive failure mechanism of backfill-encased-rock composite structures under triaxial compression—especially the synergistic mechanical effect between backfill and rock—still lacks in-depth exploration, which is exactly the core content of this study.

In this study, we carried out triaxial compression tests on BR composite structure under different confining pressures and divided the failure process into distinct stages. During the test, an AE monitoring system was used, and CT scanned the specimens in each stress stage to show the whole process of composite structure failure from the perspective of sound and vision. In the two-step stope and subsequent filling mining engineering, the BR composite structure often faces problems such as stope collapse caused by sudden instability under high confining pressure, lack of design basis for bearing capacity during the stress recovery stage, and insufficient adaptability between backfill mix ratio and confining pressure, which seriously affect mining efficiency and safety. This study improves the analysis system for the progressive failure of the backfill-rock composite structure, clarifies the sequence, stage characteristics, initial damage location, and damage evolution law of internal failure of the two media, and provides reference and theoretical support for the selection of safety factors of the BR composite structure under different confining pressures, the optimization of backfill cement-tailings ratio and solid content, and the parameter design of stope support structure.

## Experimental methods and materials

### Test materials and preparation

**Fig. 2:**
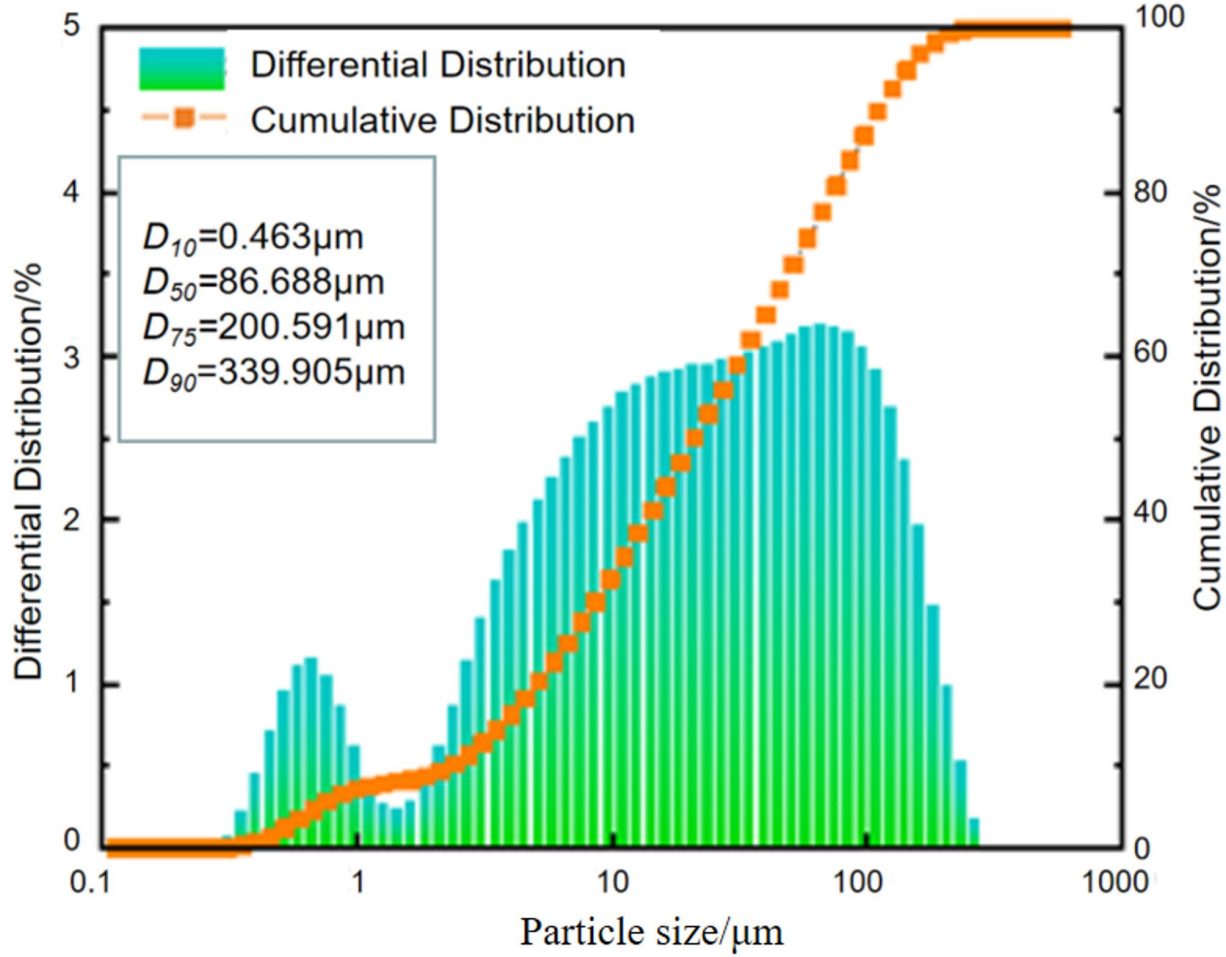
Particle size distribution curves of tailings.

The tested specimens were all 50 mm in outer diameter and 100 mm in height. The external backfill and the internal rock size ratio is 1:1. The tailings used in the filling body are taken from the full tailings of Jiaojia Gold Mine. The full tailings belong to fine-grained tailings with a median particle size of 86.69 μm and are mainly composed of quartz. The chemical composition of the tailings determined by X-ray diffraction (XRD) results are shown in Table 1, and the particle size distribution (PSD) curves are shown in Fig. 2. The cementing material is PO42.5 ordinary Portland cement, and the water is tap water. Rocks select the granite block with homogeneous texture and fewer original defects.

According to the actual engineering situation, in the backfill structure, the slurry solid content composed of cement and tailings is 70%, and the ratio of cement to tailings is 1:6. Group C of BR specimen was made, and group A of backfill-only specimen and group B of rock-only specimen for control experiment were made, with 6 specimens in each group (3 for formal test and 3 for standby).The sample size was determined based on two key considerations: first, referring to the relevant specifications for rock mechanics tests, such as GB/T 50,266 − 2013 Standard for Test Methods of Engineering Rock Mass, which recommend 3 parallel specimens for mechanical property testing to ensure data reliability; second, combining with the variability of backfill materials and the possible damage of specimens during curing and polishing, 3 standby specimens were added to avoid test failure caused by individual specimen defects. This sample size design balances test efficiency and result accuracy, ensuring that valid data can be obtained for subsequent analysis. Table 2 is the size of each group of specimens.

**Table 1:** The chemical composition of the tailings determined by XRD.

Composition	SiO_2_	Al_2_O_3_	CaO	MgO	*P*	Fe	S	Au	Ag	Cu
Content(%)	62.77	14.34	1.88	3.38	0.08	2.90	0.15	< 0.01	0.03	< 0.01

**Fig. 3:**
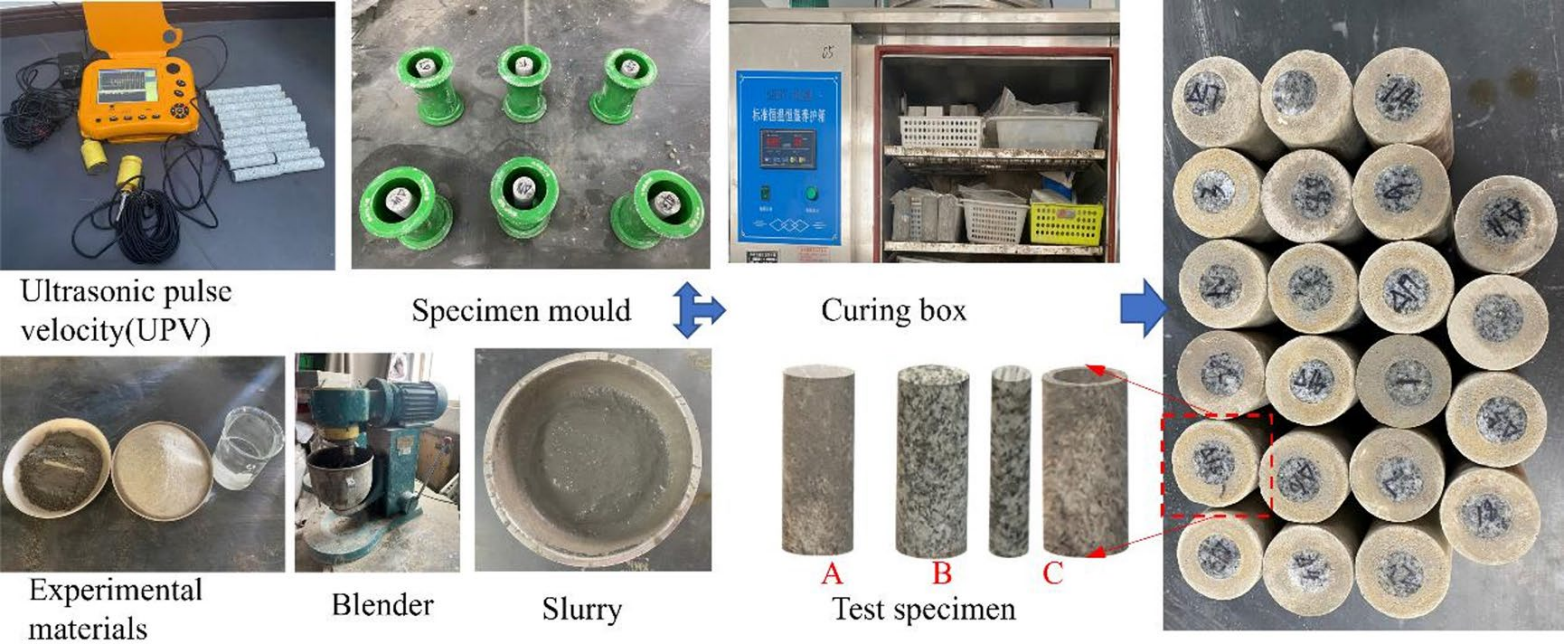
The preparation process of BR specimens.

After mixing full tailings and cement, add water and stir thoroughly to make filling body slurry. Place the granite rock in the center of the 50 × 100 mm mold and secure it with glue. Then the filling slurry is slowly poured into the mold. In order to prevent the height inconsistency between the backfill and the rock caused by the subsidence of the filling slurry, the filling slurry is continued to be poured after standing for two hours to maintain the consistency between the height of the backfill specimen and the rock. The prepared specimens were placed in YH-40B standard curing box at a constant temperature of 20 ± 5 degrees C and relative humidity of 95 ± 5% for 28 days for the experiment. After the curing, the specimens were polished and smooth. The homogeneous specimens were selected by ultrasonic pulse velocity (UPV), so as to reduce the influence of differences of specimens on the experimental results. The specimen-making process is shown in Fig. 3.

**Table 2:** Test scheme and specimen parameters.

Peer group	Size of backfill (Outer diameter*inner diameter*height/mm)	Size of rock (Outer diameter* height/mm)	Specimen size (Outer diameter* height/mm)
A	50*0*100	/	50*100
B	/	50*10	50*100
C	50*25*100	25*100	50*100

### Experimental devices and scheme design

**Fig. 4:**
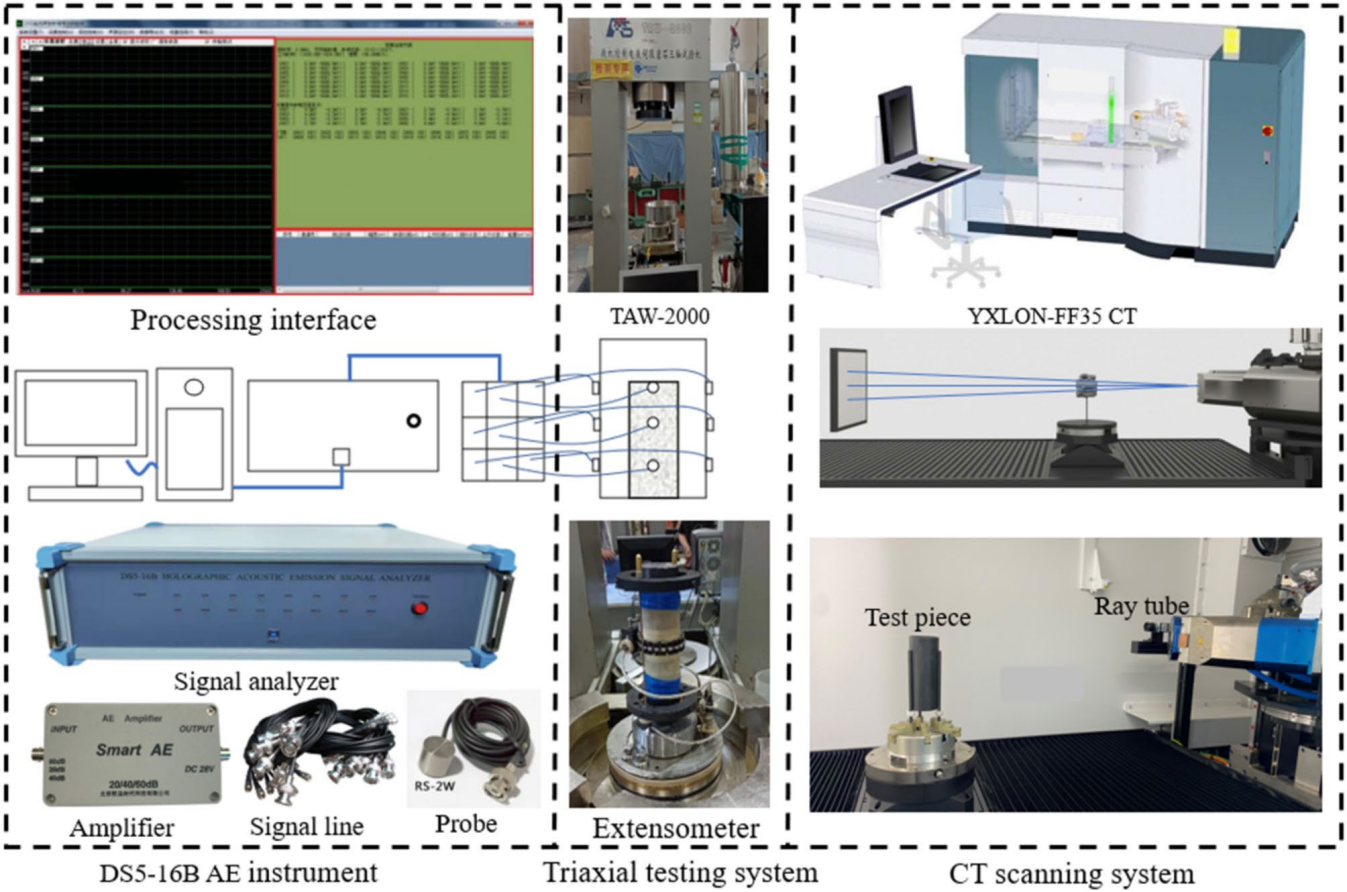
Testing apparatuses.

This experiment conducted triaxial tests on the microcomputer-controlled electro-hydraulic servo rock triaxial test machine (TAW-2000). The DS5-16B AE instrument from Softland Times Company was used for data acquisition in the compression process. Vaseline was used as a coupling agent to fix the acoustic emission probe in the triaxial outdoors. The preamplifier was 60 dB, and the threshold value was set to 100 dB to reduce the attenuation of the AE signal as much as possible and was fixed with tape. In the high position of 33,66 and 99 mm of the specimen, an AE probe was installed every 120 degrees. The compressed specimens were scanned by an industrial computed tomography system (YXLON-FF35 CT) with a resolution of 36 μm. The test apparatuses are shown in Fig. 4.

In order to analyze the stress-strain curve characteristics and macroscopic mechanical properties of BR composite structure under triaxial compression, and to obtain the failure characteristics of BR composite structure in different stages compared with rock-only specimen or backfill-only specimen. Based on previous experimental experience and preliminary test preparations, it has been demonstrated that the strength of the backfill material is relatively low. Therefore, the confining pressure for the backfill was set to 0.3 MPa, 0.5 MPa, and 0.7 MPa, respectively. For the rock and the backfill-encased-rock (BR) composite structure, the same confining pressures were selected, set at 3 MPa, 5 MPa, and 7 MPa, respectively. The confining pressures were set to 3 MPa, 5 MPa, and 7 MPa for the BR composite structure and single rock specimens, and 0.3 MPa, 0.5 MPa, and 0.7 MPa for single backfill specimens. Each group was tested three times under each confining pressure.

In the loading process of the triaxial test system, the confining pressure is first applied to the specimen by the loading method of 0.2 MPa/s constant load. When the confining pressure reaches the test value, the axial pressure is applied after 2 min. In the loading process, to effectively prevent the rockburst phenomenon when the specimen reaches the peak failure, and to observe the change of the post-peak curve, the test system and the specimen were fully fitted by the constant load loading method at first. After the pressure value in the control system increases, it is quickly changed to displacement control, with a loading rate of 0.02 mm/min. When the specimen crosses the peak value and enters the residual stage, the stress remains unchanged with the increase of strain and then the experiment is stopped.

## Results and analysis

### Stage characteristics of the stress-strain curve

The stress-strain curve can effectively reveal the strength and deformation characteristics of rock. During the triaxial compression process, the stress-strain curve visually demonstrates the deformation changes of the test specimens under different confining pressures as the pressure increases. Based on the trend of the curve, the internal volume deformation and dilatancy of the specimen, as well as the changes in original cracks and the development and propagation of new cracks, can be determined.

**Fig. 5:**
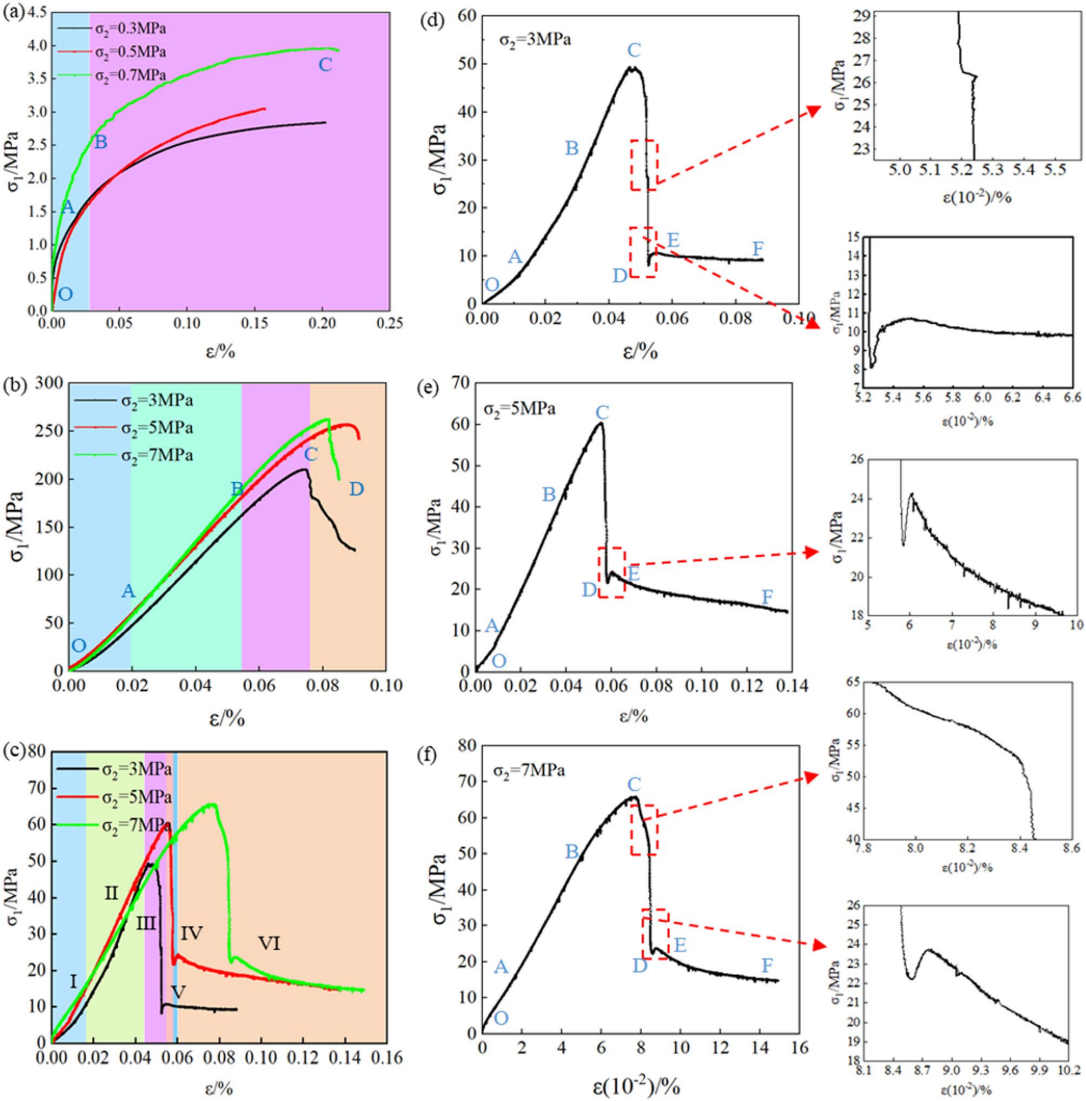
(a) Stress-strain curve of backfill; (b) Stress-strain curve of rock; (c) Stress-strain curve of BR composite structure; (d), (e), (f) Local magnifications of BR composite structure stress-strain curves under confining pressures σ_2_ = 3 MPa, 5 MPa, 7 MPa, respectively.

To investigate the mechanical property differences between the BR composite structure, single backfill, and single rock, this study conducted tests on the experimental group C and control groups A and B under different confining pressures. Figure 5 (a) and (b) present the stress-strain curves of group A (backfill-only) and group B (rock-only) under different confining pressures, respectively, while Fig. 5 (c) shows the stress-strain curve of the BR composite structure under different confining pressures. Figure 5d–f are local magnifications of the BR composite structure stress-strain curves under confining pressures σ_2_ = 3 MPa, 5 MPa, and 7 MPa, respectively.

The experimental data presented in Fig. 5 (a) demonstrate distinct rheological characteristics of the backfill material under loading conditions. The mechanical response initiates with a brief elastic deformation phase evidenced by the linear stress-strain relationship, which subsequently transitions into a non-linear deformation regime prior to exhibiting pronounced creep behavior at elevated stress levels. Detailed analysis reveals three characteristic deformation phases: (i) initial pore compaction phase (OA), (ii) non-linear yielding phase (AB) marked by microcrack formation, and (iii) final ductile failure phase (BC) characterized by macroscopic damage accumulation.In the OA stage, the external load is relatively small, and the backfill contains numerous initial pores and cracks. As the axial pressure increases, these initial pores and cracks are gradually compacted. The AB stage represents the non-linear yield stage, where the initial pores disappear after the compaction stage, and micro-cracks begin to form as the stress increases. In the BC stage, the load reaches the maximum stress value of the backfill, and internal damage occurs, leading to crack formation. At this point, the bearing capacity of the backfill no longer increases, but the strain continues to grow as the specimen deforms, resulting in creep and ductile failure.

From Fig. 5 (b), it can be seen that the rock specimen exhibits plastic-elastic behavior. In the initial loading stage, the stress-strain curve is concave. When the stress increases to a certain value, the curve enters a linear phase. As the load continues to increase, cracks begin to form inside the specimen, causing the curve to bend until the rock fails. The stress-strain curve of the rock undergoes four stages: the compaction stage (OA), the elastic-plastic stage (AB), the crack propagation stage (BC), and the post-peak failure stage (CD). In the initial loading stage (OA), the open structural planes and micro-cracks within the rock gradually close under the axial pressure. In the AB stage, the rock undergoes stable deformation as the stress steadily increases. In the BC stage, as the stress continues to rise, it reaches the yield strength of the rock, and new cracks begin to form inside the specimen. In the CD stage, a large number of cracks develop within the specimen, reaching the maximum stress value at point D, where the rock undergoes brittle failure and loses its bearing capacity.

Figure 5 (c) presents the stress-strain curves of the BR composite structure under different confining pressures. As analyzed from the figure, the peak stresses and residual stresses of the BR composite structure under the three groups of confining pressures are inconsistent. Specifically, in the elastic stage, although the stress-strain curves of the three groups of specimens all show an approximately linear trend, with the increase of confining pressure, the slope of the linear segment of the curve (i.e., the elastic modulus) becomes larger, indicating that the rock mass has a stronger elastic deformation capacity under high confining pressure. The peak stress of the specimens increases with the increase of confining pressure: the peak stress of the specimen is 48 MPa when the confining pressure is 3 MPa, and reaches 60 MPa and 66 MPa respectively when the confining pressures are 5 MPa and 7 MPa. This indicates that the greater the confining pressure, the larger the maximum axial stress that the rock mass can bear. The residual stress of the specimens also increases with the increase of confining pressure: the lower the confining pressure, the more drastic the post-peak stress drop. For example, when the confining pressure σ_2_ = 3 MPa, the stress drops rapidly to a low residual strength after reaching the peak (IV); the higher the confining pressure, the more gradual the post-peak stress decline. For instance, when the confining pressure σ_2_ = 7 MPa, the stress decays slowly after the peak (VII). This reflects the influence of confining pressure on the “ductility” of the rock mass after failure—high confining pressure makes the rock mass exhibit stronger ductility after failure–large deformation but slow stress decay, while low confining pressure makes it more brittle–stress drops sharply to residual strength.

By comparing the experimental results of control groups A and B, it can be observed that the backfill-encased-rock (BR) composite structure exhibits a stress-strain behavior similar to that of the single rock specimen before reaching the peak stress. This consistency is attributed to the rock’s dominant load-bearing role in the pre-peak stage, conforming to the classical rock mechanics theory of “stress concentration priority in high-strength media”—the rock, with higher elastic modulus and compressive strength, bears the majority of axial stress and controls the deformation trend. However, after the peak stress, the stress-strain curve of the BR composite structure shows significant differences compared to the curves of the two single-medium specimens. Specifically, when the stress drops to a certain level, it begins to rise again, which can be explained by the Mohr-Coulomb strength criterion: the backfill, as a low-strength ductile medium, provides lateral confinement to the damaged rock through its residual deformation capacity, increasing the effective confining pressure of the rock mass and thus restoring part of the bearing capacity. Furthermore, after the stress recovery, the stress-strain curve of the BR composite structure resembles that of the single backfill specimen, indicating that the backfill becomes the main load-bearing body at this stage, relying on its ductile properties to inhibit the brittle failure of the rock. Therefore, for the multi-medium composite structure of backfill and rock, its failure behavior under load differs considerably from that of either single medium. This mechanical coupling effect follows the principle of “complementary advantages between media”: the rock provides high pre-peak strength, while the backfill supplements post-peak ductility, and their interaction restricts mutual failure and deformation through interface stress transmission and lateral confinement.

Figure 5d–f respectively present the failure characteristics and local magnification of the stress-strain curves of the BR composite structure under different confining pressures. From the figures, it can be observed that the failure process of the BR composite structure can be divided into six stages: the compaction stage (OA), the elastic-plastic stage (AB), the crack propagation stage (BC), the post-peak stress decline stage (CD), the stress recovery yield stage (DE), and the ductile failure stage (EF). The characteristics of each stage are as follows:

1. Compaction stage (OA): In this stage, the initial pores and micro-cracks within both the backfill and the rock gradually disappear as the pressure increases.
2. Elastic-plastic stage (AB): In this stage, the stress and strain exhibit a linear growth trend. After the initial micro-cracks disappear, damage-induced micro-cracks begin to form.
3. Crack propagation stage (BC): Cracks begin to generate and develop in large quantities. While the stress increases slowly, the strain increases rapidly, and the composite structure specimen is gradually destroyed. When the peak point is reached, the stress decreases, and the specimen loses its bearing capacity. It is worth noting that when the confining pressure is 3 MPa, the stress fluctuation occurs after the composite structure reaches the peak point, and the stress decreases slightly at first and then continues to rise. This is due to the backfill exerts lateral pressure on it after rock failure, so that the backfill will not produce a large number of cracks in a short time and can continue to bear the pressure, so the stress rebounds. However, due to the structural damage in the rock, it is impossible to bear high stress for a long time, and it continues to decline after a short rebound.
4. Post-peak failure stress decline stage (CD): the internal crack propagation of the composite structure makes the specimen lose the bearing capacity instantaneously, and the stress decreases rapidly. When the confining pressure is 3Mpa and 7Mpa, there is a sudden increase in displacement during the decline process, indicating that the internal rock slips after the composite structure specimen reach the peak stress failure. Still, the external backfill limits the deformation of the internal rock so that the displacement only increases in a small part.
5. Stress recovery yield stage (DE): After the peak point, the stress drops to a certain level and then rises again, showing a yield stage similar to that of backfill-only specimens.
6. Ductile failure stage (EF): the stress of the composite structure no longer changes with the increase of strain, and the stress is maintained at a certain level to bear pressure and enter the ductile failure stage.

In Fig. 5 (d), the local magnification of the stress-strain curve reveals a double peak at the point of maximum stress, which is indicative of stress fluctuations during the loading process. This fluctuation implies that the specimen undergoes a certain degree of failure; however, the interaction between the backfill and rock (the two constituent media of the BR composite structure) restrains the abrupt drop in stress that would otherwise occur. Even so, under high-load conditions, the composite structure can only sustain the applied pressure for an extremely short duration before its load-bearing capacity is compromised, and the stress subsequently enters a declining phase. In the post-peak stress decline stage (CD), an unexpected stress surge is observed. When this phenomenon is compared with the post-peak failure curve of the single rock specimen, it becomes apparent that the strain of the single rock specimen increases notably after failure. In contrast, the strain surge in the BR composite structure is minimal—a difference that can be attributed to the external backfill limiting the failure-induced deformation of the internal rock.

In Fig. 5 (e), after the stress reaches its peak, it rapidly declines. In this stage, there is no sudden increase in stress, but as the stress drops to a certain level, it begins to rise again. The behavior of the stress-strain curve in this stage is consistent with that of the backfill material.

In Fig. 5 (f), during the post-peak stress decline, the initial behavior is similar to that of the rock post-peak, showing a short-term displacement surge. However, due to the interaction between the two media, the external backfill restricts the deformation of the internal rock, preventing a continuous displacement surge. After the stress drops to a certain level, the two media begin to bear the load together again, leading to a recovery in load-bearing capacity. However, due to the structural damage within the rock, it cannot continue to sustain high stress, and after a brief recovery, the stress continues to decline.

### Strength and failure characteristics of BR

**Fig. 6:**
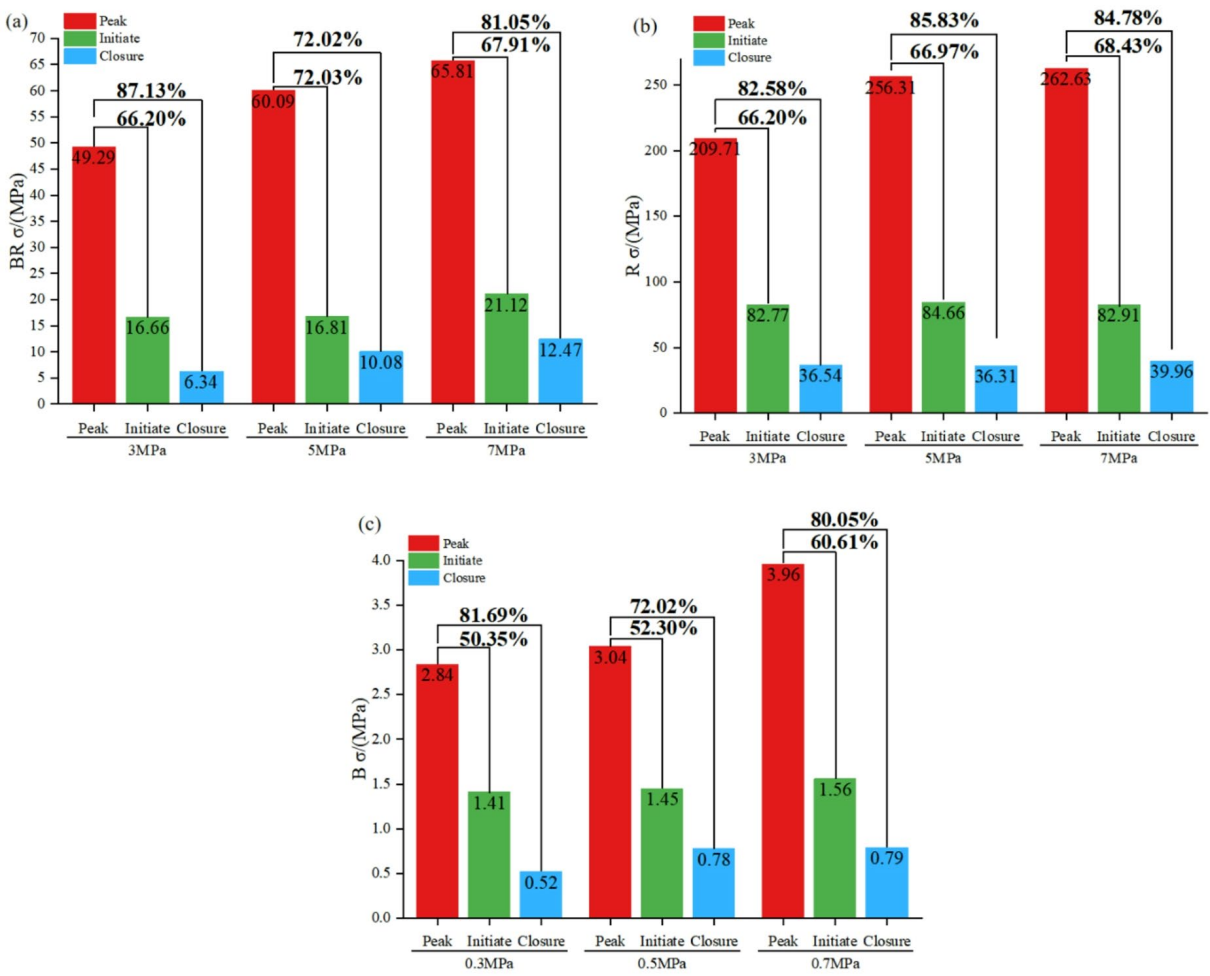
Specimen strength comparison diagram.

The pre-peak deformation stage of the specimen in triaxial compression experiments contains four important characteristic stress thresholds, and based on the stress-strain curve of the specimen, the crack closure stress (${\sigma _A}$), crack initiation stress (${\sigma _B}$), crack damage stress (${\sigma _C}$) and peak stress (${\sigma _D}$) can be determined to characterize the compressive capacity and damage of the specimen quantitatively^43^. Table 3 shows the stress values for each specimen at different compression stages. The variation of the strength of the rock specimen, BR, and the backfill is derived by analyzing the data in Table 3, as shown in Fig. 6 It can be concluded from Table 3 that the characteristic stress thresholds of each specimen at different stages of compression increase with the increase of the confining pressure, and the compressive strength increases. Figure 6 investigates the influence of confining pressure on the compressive capacity of composite structures in mining operations, in comparison to individual rock specimens. Through experimental analysis, it is observed that composite structures exhibit a greater magnitude of variation, from the yielding stage to ultimate failure, than rock-only specimens. This disparity is attributed to the encapsulation of filling materials during the microcrack propagation stage, which enhances the compressive capacity of rocks. Differ from rocks formed through long-term geological processes, fragmented components of rocks are unable to reconstitute effective load-bearing bodies during bearing failure and continued loading. Consequently, the low strength of the filling material in the composite structure facilitates rock crushing under external forces, enabling the composite structure to collectively sustain subsequent loads. Remarkably, the composite body exhibits a change value of 66.2%(3 MPa), 72.03%(5 MPa), 67.91%(7 MPa) during the transition from the yielding stage to failure, surpassing the magnitude of change observed in individual filling bodies and single rocks. Furthermore, the strength variations of the composite structure are more significant under low confining pressures (3 MPa) compared to higher confining pressures (5–7 MPa).

**Table 3:** BR threshold intensity.

NO.	Crack Closure stress (MPa)	Crack initiation stress (MPa)	Crack damage stress (MPa)	Peak stress (MPa)	Residual strength (MPa)
BR-3	6.34	16.66	41.02	49.29	9.15
BR-5	10.08	16.81	52.76	60.09	13.79
BR-7	12.47	21.12	56.49	65.81	14.93
R-3	36.54	82.77	179.08	209.71	/
R-5	36.31	84.66	209.23	256.31	/
R-7	39.96	82.91	228.12	262.63	/
B-0.3	0.52	1.41	/	2.84	/
B-0.5	0.78	1.45	/	3.04	/
B-0.7	0.79	1.56	/	3.96	/

Figure 6 presents the strength comparison of the backfill-encased-rock (BR) composite structure, single rock specimens, and single backfill specimens. By comparing Figures (a) and (b), it can be observed that the peak strength of both the BR composite structure and the single specimens increases with the rise in confining pressure. However, the influence of confining pressure on the peak strength gradually diminishes as the confining pressure increases. Specifically, when the confining pressure increases from 3 MPa to 5 MPa, the peak strength of the rock increases by 46.6 MPa, while that of the BR composite structure increases by 10.8 MPa. When the confining pressure increases from 5 MPa to 7 MPa, the peak strength of the rock increases by 6.32 MPa, and that of the BR composite structure increases by 5.72 MPa. This indicates that low confining pressure has a significant impact on the peak strength of the specimens, but as the confining pressure continues to increase, its influence on the peak strength gradually decreases, and the peak strength is ultimately determined by the specimen itself.

By analyzing Table 3; Fig. 6, it can be seen that the residual strength of the BR composite structure and the peak strength of the single backfill specimen both increase with the rise in confining pressure. The higher the confining pressure, the greater its influence on the peak strength of the backfill and the residual strength of the BR composite structure. For the backfill, when the confining pressure increases from 0.3 MPa to 0.5 MPa, the peak strength increases by 0.2 MPa, and when the confining pressure increases from 0.5 MPa to 0.7 MPa, the peak strength increases by 0.92 MPa. This further proves that under low confining pressure, the peak strength changes significantly as the confining pressure increases. For the BR composite structure, when the confining pressure increases from 3 MPa to 5 MPa, the residual strength increases by 4.64 MPa, and when the confining pressure increases from 5 MPa to 7 MPa, the residual strength increases by 1.14 MPa. This indicates that confining pressure affects the residual strength of the BR composite structure. The higher the confining pressure, the stronger the load-bearing capacity of the specimen during post-peak re-pressurization. However, as the confining pressure increases, its influence on the residual strength of the BR composite structure becomes progressively smaller.

The peak strengths of the BR specimens are still higher than that of the backfill itself compared to the backfill-only specimen. This strength enhancement under low confining pressures holds crucial implications for mining engineering operations, particularly in scenarios involving backfilling and subsequent mining activities. The alteration of the in-situ stress field caused by mining operations leads to weakened surrounding pressures in the mined out area. Strengthening pillars becomes paramount in ensuring the safety and efficiency of underground mining operations. Notably, the working face does not require high strength in uncracked ores and rocks; instead, it emphasizes the ability to sustain tunnel safety amidst natural cracks and load-induced fractures. In this regard, environmentally friendly, cost-effective, and renewable filling materials serve as effective protective barriers and should garner recognition from a broader range of experts in the field.

**Fig. 7:**
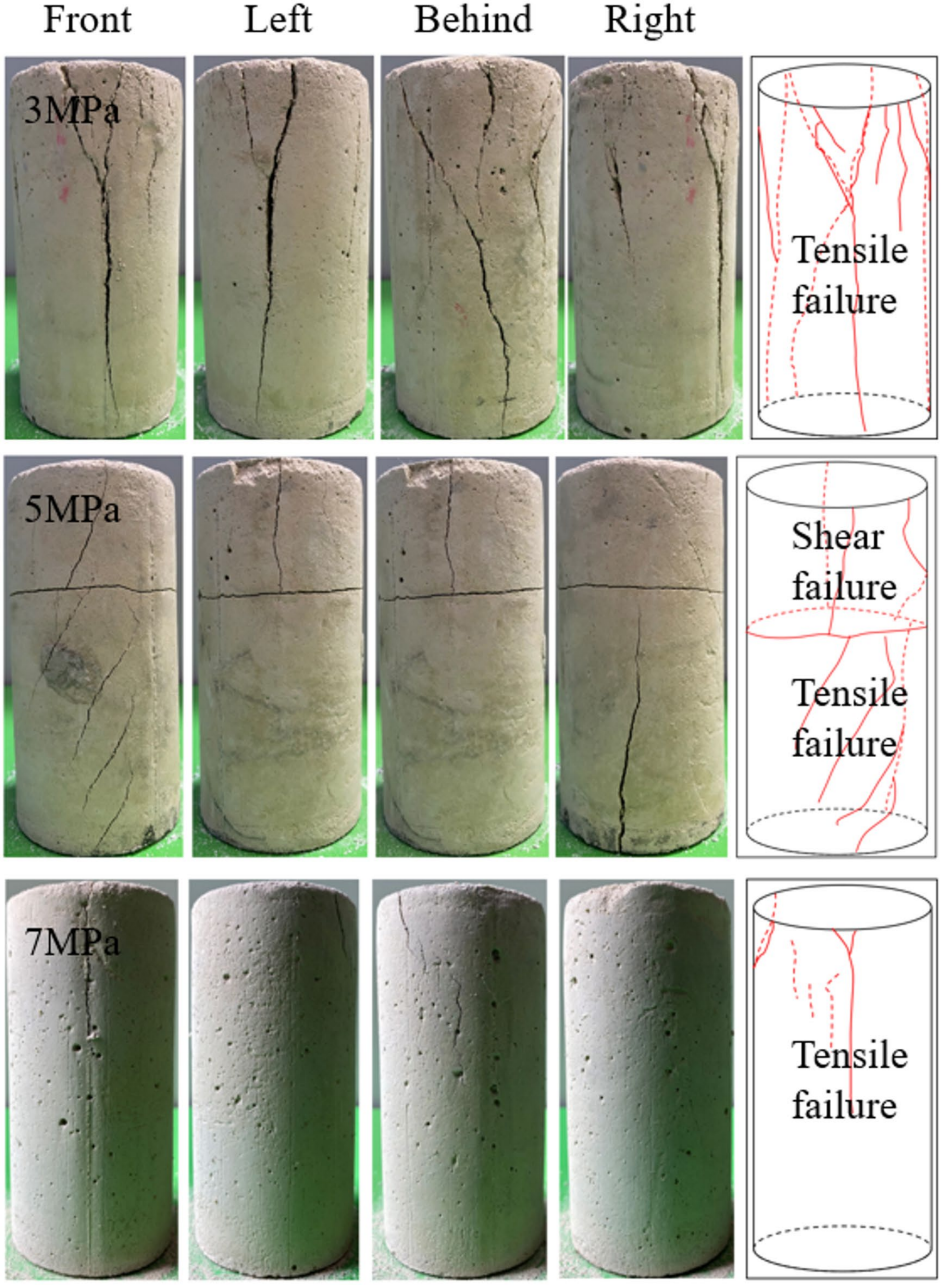
Failure mode diagram of the test piece.

Figure 7 shows the failure modes of specimens under different confining pressures. Observe the surface crack distribution of the specimens from the front, back, left and right; the figure shows the correct direction and the overall crack distribution characteristics.As can be observed from Fig. 7, the failure mode of the BR composite structure is predominantly tensile failure, and the number of cracks shows a decreasing trend as the confining pressure increases. Cracks initially originate within the internal rock. When the rock loses its load-bearing capacity and the backfill and rock start to bear the pressure collectively, the external backfill undergoes damage; eventually, the penetrating tensile failure results in the overall damage of the specimen. When cracks form in the backfill, the internal rock exerts an outward squeezing force on it. Specifically, the lower the confining pressure, the weaker the lateral constraint effect on the crack propagation of the backfill, and thus the more prominent the crack propagation phenomenon in the external backfill.

### AE characteristics

Acoustic emission (AE) can directly reflect the internal damage of BR during the evolution from deformation to failure. The relationships of the AE characteristic parameters that change with time and stress can visually express the crack initiation rate in rock at different damage development stages^44–47^. The AE hypocenter location technique can stereoscopically show the spatial location, spatial morphology, initiation rate, and propagation direction of cracks. By utilizing strain energy theory, the BR fracturing process can be completely analyzed and interpreted. The energy evolution mechanism is closely related to the micro-cracking and damage state inside the BR. AE has been proven to be one of the best techniques used to study dynamic crack propagation.

Under the action of loading, notable stress concentration arises in the vicinity of both initial cracks and newly generated cracks within the specimen, which in turn leads to a rise in the specimen’s strain energy. When the external load reaches a specific level, the defects induced by the cracks will trigger microscopic yielding or deformation. This phenomenon further causes the cracks to expand and the stress to relax. During this process, a portion of the stored energy is released, and this energy release manifests in the form of acoustic emission (AE) signals. From this mechanism, it can be deduced that acoustic emission is produced in the course of specimen loading and deformation. By analyzing AE - related parameters, including the number, amplitude, and frequency of acoustic emissions, we are able to obtain an in - depth understanding of the deformation and failure processes of the rock.

#### Variation of AE events

**Fig. 8:**
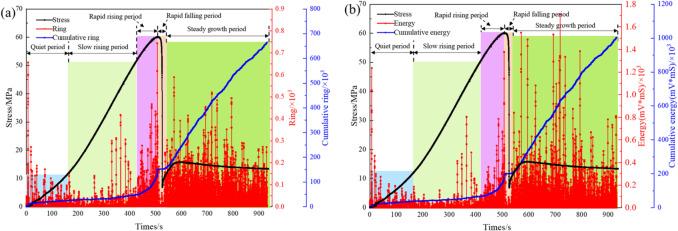
Variation of AE energy with stress.

For the BR specimens under a confining pressure of 5 MPa, we analyzed the AE counts and energy dissipation during triaxial compression based on the stress-strain curve, with the results presented in Fig. 8 As illustrated, the AE event evolution of the BR structure during triaxial compression can be divided into five stages: quiet period, slow growth period, rapid rising period, rapid falling period, and steady growth period—consistent with the naming of subsequent detailed analysis stages.

1. Quiet period (0s ~ 165s): In the compaction stage, the stress-strain curve exhibits a concave shape. As the load increases, the specimen absorbs mechanical energy from the external load. A portion of this energy is stored as elastic energy, while another portion is transferred to the closed layered planes, compacting the internal initial pore structure. The friction and sliding between the initial pores and cracks consume energy, generating acoustic emission (AE) events. During this stage, the number of AE counts and energy release signals are relatively low, with strong signals only occurring when the specimen begins to be compressed. The AE counts account for 2.8% of the entire compression failure stage, and the energy release accounts for 2.6%, indicating a quiet period of signal release.At this stage, the low AE energy and slow stress rise correspond to the compaction of initial pores rather than the generation of new cracks, so the energy consumed is mainly used for the friction and closure of existing pores rather than crack propagation.
2. Slow growth period (166–418 s): When the layered planes and initial cracks within the specimen are fully closed, the BR composite structure enters the elastic-plastic stage. During this stage, the AE signals begin to gradually increase and strengthen. The external energy input in this phase is primarily used for elastic compaction, with most of the energy being converted into accumulated elastic strain energy. The AE counts account for 4.5% of the entire compression failure stage, and the energy release accounts for 3.9%, indicating a slow growth period of signal release.The gradual increase in AE energy and linear growth of stress in this stage are related to the initial initiation of micro-cracks (not yet propagated in large quantities), and the stored elastic strain energy is slightly released with the formation of small micro-cracks.
3. Rapid rising period (419–507 s): In the unstable crack propagation stage, the load gradually approaches the elastic limit strength of the specimen. A large number of cracks are generated within the BR composite structure, leading to a rapid increase in dissipated energy over a short period. The AE counts sharply rise to their peak, accounting for 15.3% of the entire compression failure stage, and the energy release accounts for 13.2%.The sharp surge in AE energy and the approaching peak stress are directly caused by the massive initiation and interconnection of internal cracks, as the accumulated elastic strain energy is rapidly released during the unstable propagation of cracks.
4. Rapid falling period (508s ~ 535s): When the BR composite structure fails, its pressure bearing capacity is rapidly lost in a short time. Meanwhile, the internal structure of the BR gets damaged, making it unable to sustain external stress any longer. During this stage, the residual energy stored inside the structure is released, and the number of AE counts drops to nearly zero.The sudden drop in stress and the rapid decline of AE energy to near zero are due to the complete loss of the specimen’s load-bearing capacity after macro-crack penetration, and no new cracks are generated to trigger additional energy release.
5. Steady growth period (536–936 s): In this stage, a large amount of AE counts is generated and energy release increases. With the continuous release of energy in BR ductile failure, the compressive capacity of BR is maintained at a certain level during energy release. The AE counts accounts for 76.5% of the whole compression failure stage, and the energy release accounts for 79.5%.The stable high AE energy and stable residual stress in this stage correspond to the continuous friction and slight expansion of existing cracks rather than the formation of new macro-cracks, and the backfill and damaged rock together bear the load to maintain the residual compressive capacity while releasing energy continuously.

#### Spatio-temporal evolution of AE events

**Fig. 9:**
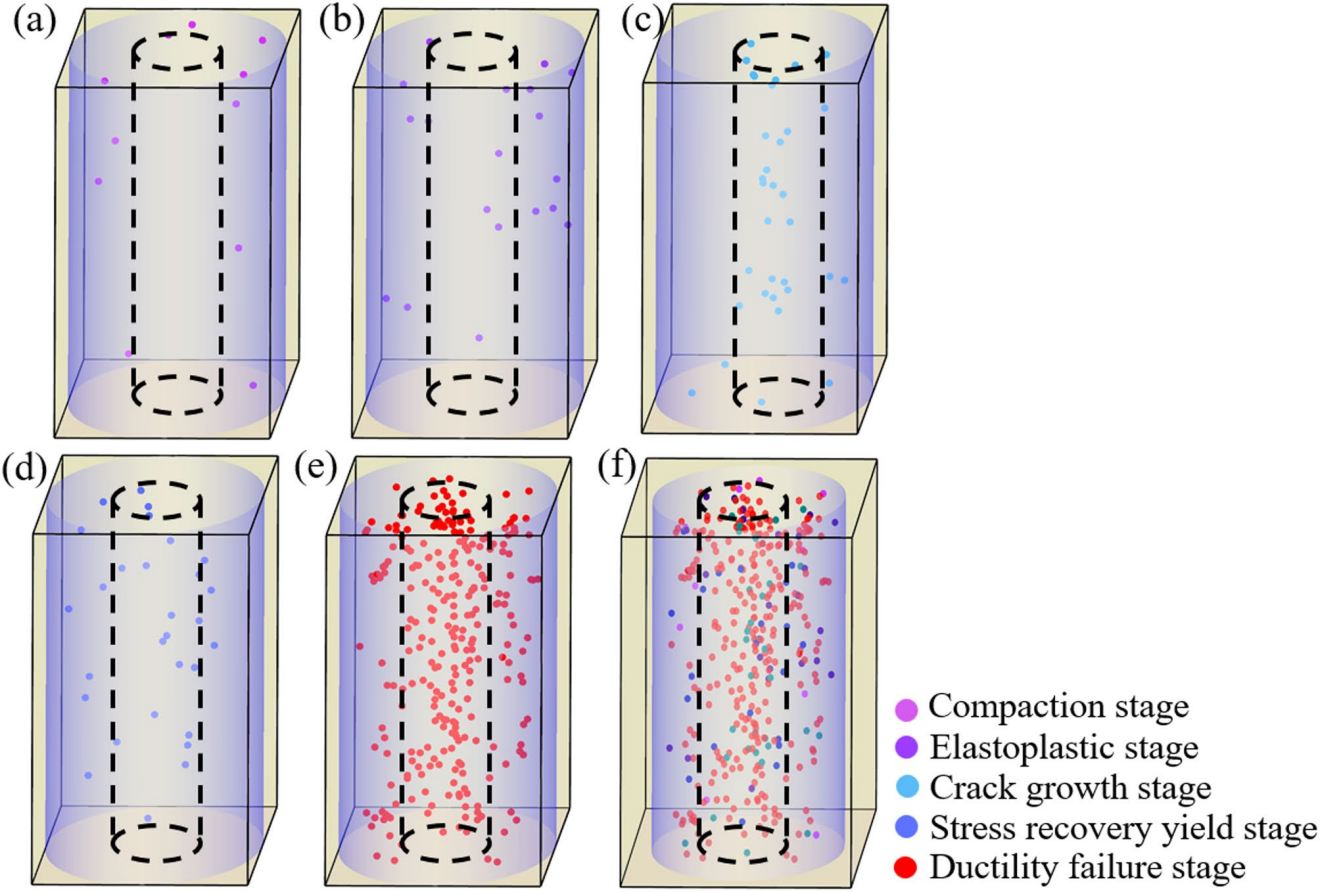
Spatio-temporal evolution of AE events during different strain intervals.

As shown in Fig. 9, the spatio-temporal variation of AE events of BR specimens under the confining pressure of 5MP. Fig. (a)~(e) are AE event distributions at each failure stage of BR, and Fig. (f) is AE event distribution during the entire failure process. From the figure, it can be observed that in the initial loading stage, AE events are predominantly active in the external backfill. As the pressure increases and the rock gradually undergoes failure, a significant number of AE events occur within the rock. In the final stage, when the specimen fails, both the backfill and the rock simultaneously generate a large number of AE signals. The evolution pattern of AE events is closely related to the energy conversion law in rock mechanics—AE signals are the external manifestation of internal strain energy release during material damage, and the intensity and frequency of signals correspond to the rate and scale of crack propagation. The stage characteristics are as follows:

1. Compaction Stage (OA): Acoustic emission (AE) events are generated in the external backfill, with signals primarily concentrated at the two ends of the backfill and fewer signals in the middle region. No changes are observed inside the rock. According to the energy conservation principle, the external load energy is mainly consumed by the compaction of initial pores and micro-cracks in the backfill, and the low AE energy release indicates that the energy is converted into frictional energy between pore walls rather than crack propagation energy. The rock, with high compactness, hardly undergoes pore compaction, so no AE signals are generated. During this stage, the initial pores and micro-cracks within the backfill are gradually compacted.
2. Elastic-Plastic Stage (AB): The number of AE events in the backfill increases significantly, with a small number of signals appearing at the interface between the rock and the backfill. Sparse signals begin to emerge inside the rock.
3. Crack Propagation Stage (BC): AE events inside the rock continue to increase, while AE events in the external backfill decrease. As the load gradually increases, the pores in the external backfill are compacted, and the internal backfill becomes the main load-bearing structure. Cracks begin to develop inside, and as the stress continues to rise, these cracks propagate until the entire specimen structure is damaged. Under high stress, the internal rock fails, and the external backfill, due to its weaker load-bearing capacity, also fails simultaneously.
4. Post-Peak Stress Decline Stage (CD): The backfill-encased-rock (BR) composite structure loses its load-bearing capacity in a short time, and no AE events are generated. Therefore, this stage is not shown in Fig. 9.
5. Stress Recovery Yield Stage (DE): The BR composite structure begins to bear load again, and a large number of AE events are generated inside the rock, while only a few AE events occur in the external backfill. During this stage, the numerous cracks inside the rock rub against each other under the load, preventing further structural damage and releasing a large number of signals.
6. Ductile Failure Stage (EF): A large number of AE events are generated both in the backfill and inside the rock. This indicates that during this stage, the cracks inside the rock continue to rub against each other, preventing further damage, while the cracks in the external backfill develop and begin to interact with the rock. As the backfill restricts the deformation and failure of the internal rock, the development of its own cracks releases signals.

**Fig. 10:**
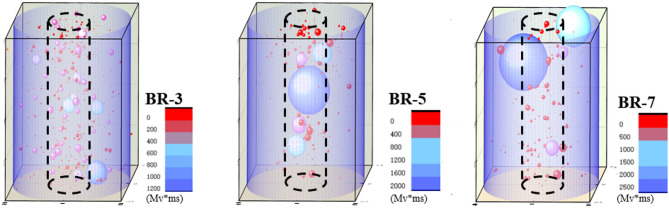
Energy dissipation distribution map.

Figure 10 shows the energy dissipation distribution of the BR composite specimen during the triaxial compression process. The color gradient from red to blue represents the magnitude of dissipated energy, with red indicating lower energy and blue indicating higher energy. The distribution also visually indicates the regions where the maximum energy dissipation occurs during the compression failure process. From the figure, it can be observed that as the confining pressure increases, the dissipated energy during the failure of the BR composite structure also increases. This indicates that with the increase in confining pressure, the peak strength of the specimen increases, and the external mechanical energy input rises. Consequently, the specimen absorbs more energy internally. During the process from initial energy absorption to later crack propagation and energy dissipation, the energy remains balanced.The energy release in the external backfill is significantly lower than that in the rock region, but there are many energy release points, indicating that the external backfill contains numerous initial pores and generates many micro-cracks during the failure process. In contrast, the internal rock releases a large amount of energy, indicating that during the loading process, the internal rock acts as the primary load-bearing structure, absorbing the majority of the mechanical energy. During failure, the maximum energy release occurs at the interface between the backfill and the rock. This suggests that when the specimen is about to undergo deformation and failure, cracks form inside the rock and propagate outward. At this point, the external backfill restricts the mechanical energy released by the outward propagation of the internal rock. The backfill is not fully penetrated by the mechanical energy released from the rock failure, and as a result, a significant amount of energy is released at the interface between the backfill and the rock.

### Evolution characteristics of micro-fracture under computed tomography

**Fig. 11:**
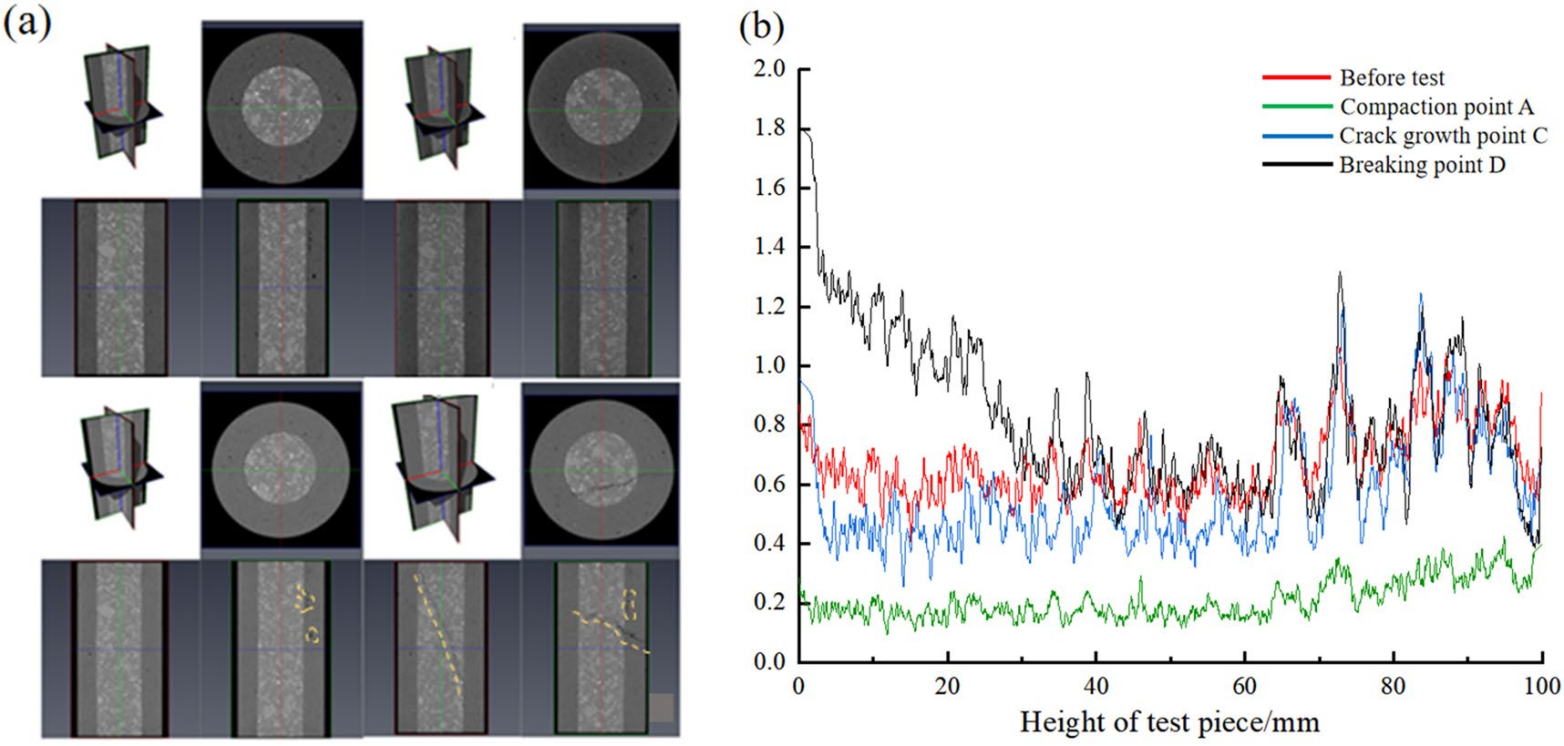
CT scan at various stages during the triaxial test. (a)Typical 2D slice of CT scan at each stage during the triaxial test, (b)Porosity at each stage during the triaxial test.

As shown in Fig. 11 (a), it is a two-dimensional slice diagram of the CT scan results in X, Y and Z directions at each stage of the triaxial compression process of the BR specimen. It can be seen from the diagram that there are a large number of initial pores in the external backfill and the internal rock structure is complete. During the compression process, cracks developed less before reaching the peak point, and most of the same characteristics still existed, without obvious deformation or fracture. After the specimen was damaged at the peak point, shear failure occurred, resulting in large cracks penetrating through the rock and backfill.

Figure 11 (b) illustrates the changes in porosity of the BR composite structure at various stages during the compression process. The red curve represents the porosity of the specimen before the test, i.e., the initial porosity. The green curve indicates the porosity at the end of the compaction stage, the blue curve shows the porosity at the end of the elastic-plastic stage, and the black curve represents the porosity at the peak failure point. By comparing these curves, it can be observed that the porosity is the smallest during the compaction stage, as the initial pores disappear with the increase in pressure. After the elastic-plastic stage, the specimen develops damage-induced micro-cracks, leading to an increase in porosity. However, the porosity at this stage is still lower than the initial porosity, indicating that the micro-cracks generated within the specimen increase the porosity, but the volume of these micro-cracks is smaller than that of the initial cracks. After the crack propagation stage, the porosity reaches its maximum. At this point, the specimen undergoes internal failure, resulting in large cracks. The porosity is highest at the ends of the specimen, indicating that most of the damage occurs at the ends. In contrast, the porosity is lowest in the middle of the specimen, suggesting that the central region experiences the least damage and acts as a weakened zone during failure.

**Fig. 12:**
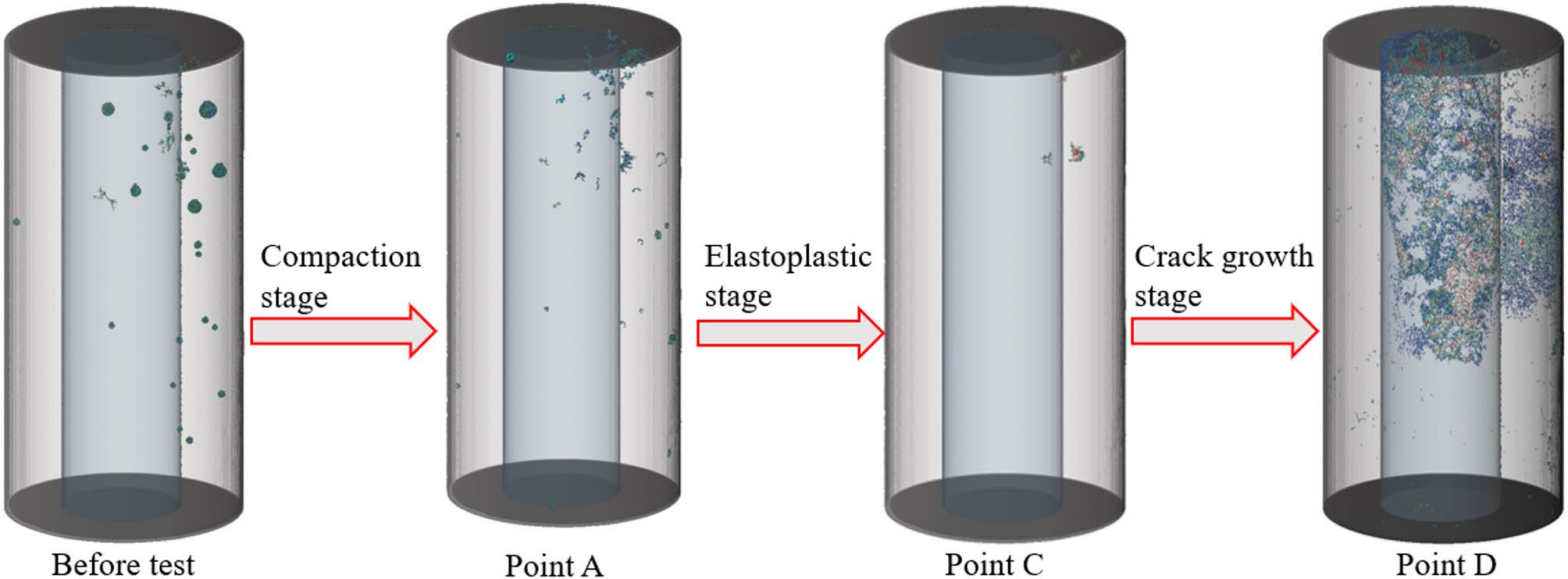
A 3D crack evolution at each stage point.

Figure 12 shows the joint’s crack growth diagram at each failure stage during BR compression.By comparing the three-dimensional crack evolution at each stage, it can be concluded that as the load gradually increases, the initial pores in the backfill region gradually disappear. After the specimen reaches the peak failure point, penetrating cracks develop in the rock.

Before the test, the backfill region contained numerous pores of varying sizes, along with a few micro-cracks, while the rock interior was dense and free of micro-cracks.

At point A, after the compaction stage, the smaller initial pores completely disappeared, and the larger initial pores were compressed into micro-cracks. No new cracks were generated during this stage.

At point B, after the elastic-plastic stage, the initial pores within the backfill completely disappeared. Under high stress, localized damage began to occur, resulting in the formation of micro-cracks.

At point C, after the crack propagation stage, the cracks inside the specimen, which began to develop at point B, reached point C, where the specimen reached its failure point. Penetrating cracks formed, extending from the rock to the backfill. This crack propagation path conforms to the maximum tensile stress theory in rock mechanics: when the internal tensile stress of the rock exceeds its tensile strength, cracks initiate and propagate along the direction perpendicular to the maximum tensile stress. Based on the crack distribution, the failure of the specimen is characterized as tensile failure. The rock cracked from the upper end and propagated to the middle, but the lower part of the rock remained intact—this is due to the mechanical coupling effect at the backfill-rock interface: the backfill restricts the lateral expansion of the rock through interface adhesion and friction, reducing the stress concentration at the lower part of the rock and inhibiting further crack propagation. The cracks extended into the backfill, where they connected with the cracks in the rock, indicating that the cracks originated within the rock and propagated into the backfill. The backfill, with lower tensile strength, undergoes secondary cracking under the tensile stress transmitted by the rock cracks, forming a continuous fracture system.

Additionally, the backfill on the side of the rock that did not fail also showed no cracks, further confirming that the cracks in the backfill resulted from the propagation of internal rock cracks. The lower end of the rock did not fail, which is significantly different from the failure mode of a single rock specimen. In the failure process of a single rock specimen, cracks propagate from the upper end to the bottom, causing damage throughout the entire rock. However, when the rock and backfill form a combined load-bearing structure, the cracks propagate from the rock into the backfill, preventing complete failure of the rock structure. At this point, the specimen undergoes deformation failure. Since the external backfill does not experience structural failure, the cracks in the backfill are caused by the dilation of the internal rock. Therefore, the external backfill begins to protect the internal rock from further damage when the rock can no longer withstand high stress. On one hand, it creates a hoop effect, preventing further propagation of rock cracks, and on the other hand, it combines with the residual stress of the rock to bear the load again, causing the stress of the specimen to rise once more.

**Fig. 13:**
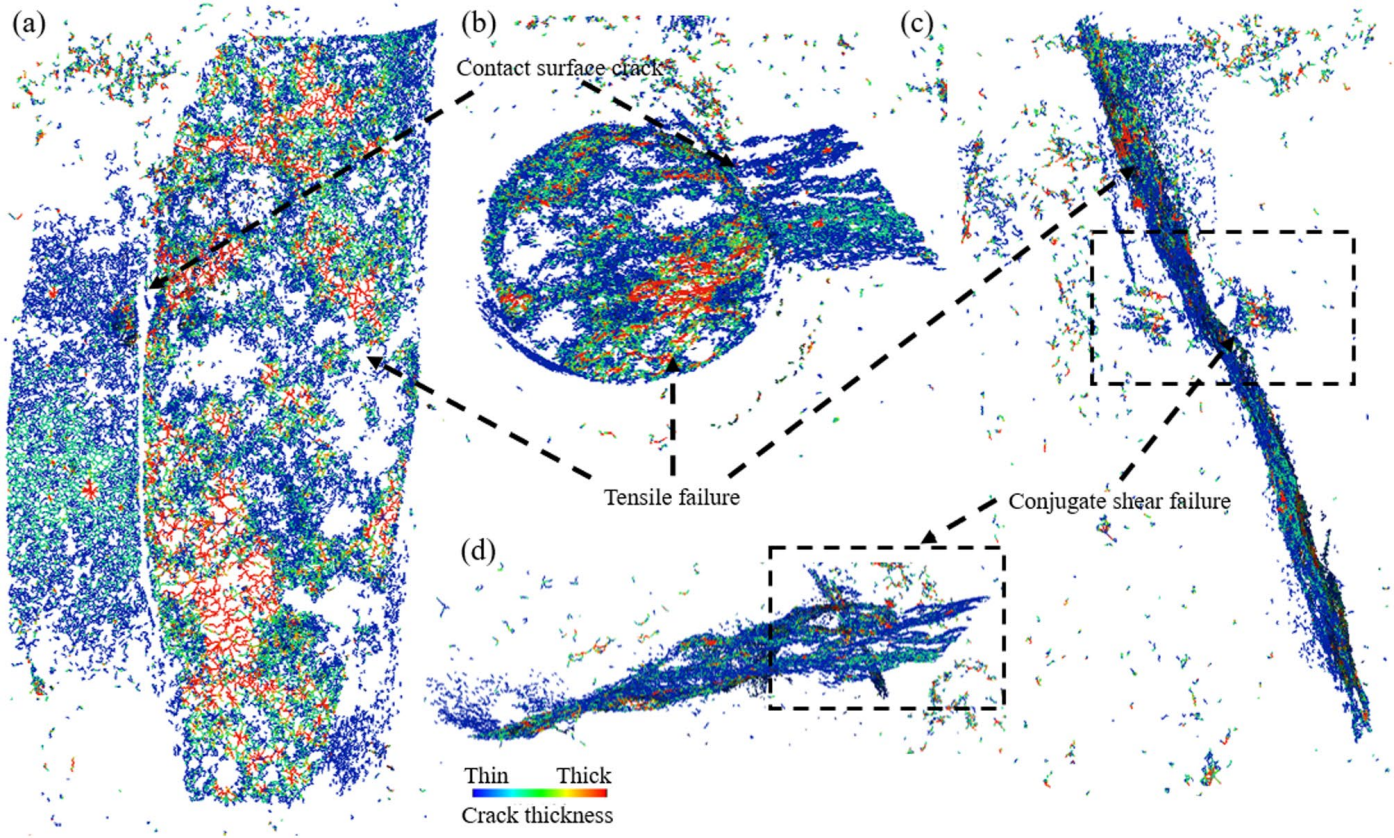
Post-failure crack skeletonization. (a) Main view, (b) Top view, (c) Left view, (d) Local view.

Figure 13 shows the results of the crack skeleton of the BR specimens destroyed. A is the main view, B is the top view, C is the left view, and D is the local view. The color from blue to red represents the thickness of the crack from thin to thick. It can be seen from the various views that BR is mainly a tensile failure, diagonally downward failure from the periphery of the rock end, separation occurs at the contact surface between backfill and rock, and a small area of conjugate shear failure occurs. From the analysis of the cracks, it can be observed that the darker-colored areas are distributed within the rock, indicating that the cracks in the internal rock are larger and propagate in stages. In contrast, the external backfill exhibits lighter-colored areas, suggesting that the cracks are smaller and the degree of damage to the backfill is relatively low.

## Discussion

Compared with backfill/rock only specimens failure processes, the BR structure shows different failure laws. Based on the AE and CT technology used in the compression process, the BR failure process is analyzed from the two perspectives of acoustics and vision. The interaction law between backfill and rock during the failure process is as follows: before the peak failure point, the stress-strain curve characteristics of BR and rock are similar, and they go through the compaction stage, elastc-plastic stage and crack growth stage. The failure process of BR composite structure is mainly determined by rock before BR reaches the peak point because rock is the main factor to bear pressure. After the peak failure point, the stress decreases rapidly, but the bearing capacity is not lost. When the pressure drops to a certain level, the stress strain curve is similar to that of the backfill.

Compared with existing studies on backfill-rock composite structures, the six-stage deformation-failure process of the BR composite structure proposed in this study—including compaction stage, elastic-plastic stage, crack propagation stage, post-peak stress decline stage, stress recovery yield stage, and ductile failure stage—aligns with the findings of Ru et al.^41^ in confirming that the backfill-rock interface exerts a regulatory effect on failure behavior. However, a distinction exists: this study further identifies the “stress recovery yield stage,” which received limited emphasis in Ru et al.’s research that focused primarily on interface characteristics. This discrepancy stems from the fact that the present study centers on the overall mechanical response of the composite structure under triaxial compression, whereas Ru et al.^41^ mainly investigated the microscale interface shear behavior.

Additionally, Song et al.^23^ previously reported the stress-strain curve characteristics of surrounding rock-backfill combined structures. In comparison, the BR composite structure in this study exhibits higher residual strength under the same confining pressure. For instance, at a confining pressure of 3 MPa, the residual strength of the BR composite structure in this study is 9.15 MPa, while the corresponding value reported by Song et al. is approximately 6 MPa. This difference is attributed to the 1:1 size ratio of backfill to rock adopted in this study—a ratio that enhances the synergistic load-bearing effect of the two media—whereas Song et al.^23^ used an unequal size ratio for backfill and rock.

During the compaction stage, AE counts are low, and the energy is low. At this stage, the AE events mainly occur in the backfill. At the same time, the BR porosity decreases, the initial pore of the backfill gradually disappears, and there are still micro-cracks, which indicates that the external backfill changes greatly during the compaction stage. The internal pores disappear as the pressure increases, but due to the strong pressure-bearing capacity of the internal rock, the backfill is in the non-linear yield stage.

In the elastic-plastic stage, AE enters a slow growth period, initial micro-cracks disappear in the backfill, AE counts and energy release increase, and a few AE events occur in internal rock. At this time, BR porosity increases, indicating that with the pressure increase, initial micro-cracks are compacted, and new cracks are generated. At the same time, the external backfill enters the ductile failure stage.

After entering the crack propagation stage, the AE signal increases rapidly. At this stage, AE events mainly occur inside the rock, while the external backfill remains almost unchanged. It can be seen from CT scanning results that there are large cracks penetrating throughout rock and backfill in BR. The BR’s porosity increases, indicating that the BR’s crack extends from rock to backfill before peak failure. Then the external backfill is destroyed after the rock loses its pressure-bearing capacity.

At the stage of post-peak failure stress decrease, BR undergoes structural damage and loses its pressure-bearing capacity momentarily. During the process of stress decrease, only BR internal residual energy is released, resulting in a few AE events. At the same time, the displacement surge occurs in the process of stress reduction, but it is smaller than that of a rock-only specimen, because the external filling body obstructs the damage of the internal rock.

After reaching the lowest point, the stress value rises again and enters the stress recovery yield stage, at which time AE events increase substantially. According to the anchor point of acoustic emission events, there are more AE events inside the rock at this stage, indicating that the pressure is mainly borne by the inner rock.

As the pressure increases, it enters the ductile failure stage. For the whole post-peak stress recovery stage, the AE event is at a relatively high level, and the high release of energy indicates that BR still has the bearing capacity at this time. While the energy is released, the bearing capacity of BR remains at a certain level, indicating that after the internal rock failure, the backfill and the damaged rock bear pressure together. The continuous failure of rock releases more energy, and the elastic-viscous properties of the backfill maintain the compressive capacity of BR at a certain level with the help of the compressive capacity of rock.

It should be noted that this study has certain limitations: the triaxial compression tests of the BR composite structure were conducted in a controlled indoor environment with a confining pressure range of 5–20 MPa, which does not fully cover the complex on-site conditions; the detection mainly relies on triaxial tests, AE monitoring, and CT scanning, leading to a slight insufficiency in the real-time quantitative analysis of internal stress transmission. In subsequent research, we will optimize experimental parameters, integrate environmental simulation, and combine numerical simulation to improve detection methods, so as to better align with engineering practice.

## Conclusions

This study investigates the mechanical behavior, energy evolution, and micro-fracture characteristics of the backfill-encased-rock (BR) composite structure under triaxial compression. It combines triaxial compression tests, acoustic emission monitoring, and computed tomography scanning to clarify the synergistic mechanical interaction between backfill and rock, providing actionable insights for stope stability design in underground metal mining. The key conclusions are as follows:

1. The BR composite structure shows a unique 6-stage deformation-failure process under triaxial compression, different from the 3-stage behavior of single backfill (compaction, non-linear yielding, ductile failure) and 4-stage behavior of single rock (compaction, elastic-plastic, crack propagation, post-peak brittle failure). The 6 stages are compaction, elastic-plastic, crack propagation, post-peak stress decline, stress recovery yield, and ductile failure. Pre-peak deformation and failure are dominated by the rock, which acts as the primary load-bearing component, while post-peak residual bearing capacity is sustained by the backfill. This load-sharing mechanism reflects the collaborative interaction between the two media, which cannot be captured by single-medium studies.
2. A spatiotemporal correlation between acoustic emission energy evolution and computed tomography-observed pore/crack dynamics is established for the first time. The acoustic emission energy evolution of the BR structure is divided into five stages: quiet period, slow growth period, rapid rising period, rapid falling period, and steady growth period. Among these, 79.5% of the total acoustic emission energy is released during the steady growth period, which corresponds to the ductile failure stage of the BR structure. This observation is consistent with computed tomography findings of continuous friction and slight expansion of existing cracks. Computed tomography scanning further reveals the spatial origin and propagation path of cracks: cracks first initiate inside the rock, then propagate toward the backfill, and the backfill-rock interface serves as a key constraint zone. It restricts rock crack expansion through lateral confinement and enables partial recovery of bearing capacity in the post-peak stage by collaborating with damaged rock.
3. Under confining pressures of 3–7 MPa (tested in 3 groups of parallel experiments), the peak strength of the BR composite structure is 20%–30% lower than that of single rock specimens. For example, at 3 MPa confining pressure, the BR structure has a peak strength of 49.29 MPa while the single rock has 209.71 MPa. In contrast, the residual strength of the BR composite structure is 65%–80% higher than that of single backfill specimens; at 3 MPa confining pressure, the BR structure has a residual strength of 9.15 MPa while the single backfill has no measurable residual strength. Confining pressure enhances both the peak strength and energy absorption capacity of the BR structure. As confining pressure increases from 3 MPa to 7 MPa, the peak stress of the BR structure rises from 49.29 MPa to 65.81 MPa, and energy dissipation at the backfill-rock interface—the primary energy release zone—increases significantly. For the fine-grained tailings (median particle size 86.69 μm) used in this study, a cement-tailings ratio of 1:6 with 70% solid content balances strength and cost-effectiveness, providing a reference for backfill parameter optimization. Additionally, a safety factor range of 1.2–1.5 is recommended for BR structures under 3–5 MPa confining pressure.
4. The post-peak stress recovery ability of the BR composite structure, enabled by the synergistic effect between backfill and rock, can reduce the risk of sudden stope collapse under high confining pressure. This provides practical guidance for stope stability design in two-step stope post-backfill mining. This study has limitations: experiments were conducted on homogeneous granite and a fixed backfill ratio, and did not account for non-homogeneous rock masses, dynamic loads such as blasting-induced vibrations, or long-term creep effects. Future research should validate the applicability of these conclusions under complex engineering conditions to further refine the BR failure model.

## Data Availability

Data will be made available on request.Data can be requested from the corresponding author (Ziyi Zeng, E-mail: M202410090@xs.ustb.edu.cn) with a reasonable research purpose.
